# DNA-encoded dynamic hydrogels for 3D bioprinted cartilage organoids

**DOI:** 10.1016/j.mtbio.2025.101509

**Published:** 2025-01-21

**Authors:** Ziyu Chen, Hao Zhang, Jingtao Huang, Weizong Weng, Zhen Geng, Mengmeng Li, Jiacan Su

**Affiliations:** a Institute of Translational Medicine, Shanghai University, Shanghai, 200444, China; b Musculoskeletal Organoid Research Center, Shanghai University, Shanghai, 200444, China; c School of Medicine, Shanghai University, Shanghai, 200444, China; d Department of Orthopedics, Shanghai Zhongye Hospital, Shanghai, 201900, China; e Sanming Institute of Translational Medicine, Fujian, 365004, China; f Department of Orthopedics, Xinhua Hospital, Shanghai Jiao Tong University School of Medicine, Shanghai, 200092, China

**Keywords:** DNA hydrogel, Bioprinting, Cartilage organoids, Tissue engineering

## Abstract

Articular cartilage, composed of chondrocytes within a dynamic viscoelastic matrix, has limited self-repair capacity, posing a significant challenge for regeneration. Constructing high-fidelity cartilage organoids through three-dimensional (3D) bioprinting to replicate the structure and physiological functions of cartilage is crucial for regenerative medicine, drug screening, and disease modeling. However, commonly used matrix bioinks lack reversible cross-linking and precise controllability, hindering dynamic cellular regulation. Thus, encoding bioinks adaptive for cultivating cartilage organoids is an attractive idea. DNA, with its ability to be intricately encoded and reversibly cross-linked into hydrogels, offers precise manipulation at both molecular and spatial structural levels. This endows the hydrogels with viscoelasticity, printability, cell recognition, and stimuli responsiveness. This paper elaborates on strategies to encode bioink via DNA, emphasizing the regulation of predictable dynamic properties and the resulting interactions with cell behavior. The significance of these interactions for the construction of cartilage organoids is highlighted. Finally, we discuss the challenges and future prospects of using DNA-encoded hydrogels for 3D bioprinted cartilage organoids, underscoring their potential impact on advancing biomedical applications.

## Graphical abstract

**Figure:**
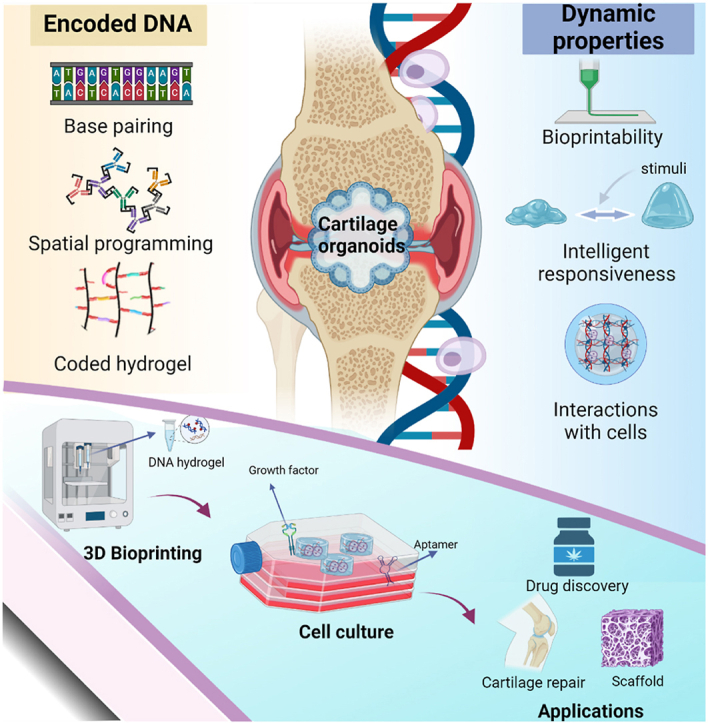

## 1 Introduction

Articular cartilage, an avascular and non-neural connective tissue derived from the mesoderm, provides elasticity and toughness essential for joint protection and movement [1,2]. Despite these attributes, its limited capacity for self-repair due to the lack of vascular and neural support leads to significant challenges when cartilage is damaged or degraded [3]. Such damage often results in joint pain, functional impairment, and osteoarthritis (OA), a condition affecting approximately 250 million people globally [4]. The economic burden of OA, including medical expenses and income loss, amounts to approximately 303 billion dollars annually [5]. Current treatment strategies for cartilage repair include microfracture, autologous chondrocyte implantation, and allogeneic/autologous cartilage transplantation [6]. However, these methods have limitations: microfracture can result in fibrocartilage rather than hyaline cartilage, while cartilage transplantation is hindered by issues such as donor shortages, poor integration, and surgical complications [7,8]. Therefore, the development of more effective, reliable, and sustainable strategies for cartilage regeneration is of paramount importance.

Recent advancements in tissue engineering have focused on creating organoids that mimic native tissues via in vitro culture techniques. This approach has gained traction for generating organoids of various tissues, including cartilage, by leveraging specific stem cells cultured in 3D environments to form functional tissue clusters for regenerative applications, drug screening, and disease modeling [9,10]. Cartilage organoid construction generally follows two approaches: scaffold-free self-assembly and scaffold-based co-culture. Scaffold-free self-assembly benefits from high biocompatibility and accurate tissue simulation but suffers from weaker mechanical properties and difficulties in maintaining stable 3D structure [11,12]. Conversely, matrix based methods provide physical support that enhances cell adhesion, proliferation, and microenvironment control. Early efforts focused on using cultured chondrocytes and conventional hydrogel matrix such as collagen, hyaluronic acid, and polyethylene glycol (PEG) to reconstruct 3D cartilage tissue [13,14].

With the progression of tissue engineering, the development of more sophisticated 3D cultures has been facilitated by the utilization of advanced hydrogel matrices to create scaffolds that more accurately replicate the mechanical and biochemical properties of natural cartilage [[15], [16], [17]]. Concurrently, the emergence of 3D bioprinting technology has revolutionized organoid construction, enabling precise control over the spatial arrangement of cells and biomaterials, and facilitating the fabrication of complex, multi-layered structures that are more representative of in vivo tissues [18]. Despite these advancements, constructing cartilage organoids via bioprinting remains challenging [19].

Organoid culture differs fundamentally from traditional tissue engineering by involving dynamic and reciprocal interactions between cells and the bioactive factors within the extracellular matrix (ECM) (Table 1). Therefore, the development of bioinks for 3D bioprinting, particularly for cartilage organoids, requires materials that are not only printable but also possess tunable physical and chemical properties that can emulate the dynamic mechanical environment of natural cartilage. This includes the ability to adjust the elastic modulus and dynamic responsiveness to cellular and environmental cues, which are crucial for mimicking the homeostasis and repair mechanisms of native cartilage [20,21]. Traditional hydrogels used as bioinks, such as Matrigel®, often lack the reversible cross-linking and precise tunability required for these applications, limiting their effectiveness in organoid culture and bioprinting [13,22,23].

**Table 1:** Comparison of cell-encapsulated hydrogels and cartilage organoids.

Characteristics	Cell-encapsulated hydrogel	Cartilage Organoids
Culture Time	Short	Long
Physiologic/pathologic representation	Limited	Semiphysiologic/Semipathologic
Structural representation	Customizable structures through 3D bioprinting	Customizable structures through 3D bioprinting
Aims	Providing adhesion sites, protecting cells, inducing specific behaviors	Fabricating 3D Cartilage tissue in vitro for regeneration, drug screening and disease modeling
Key features	Biocompatobiliy, well defined biochemical and mechanical properties	Production of ECM similar to physiologic or pathologic tissue.
Refs	[24,25]	[26,27]

DNA, with its capacity for intricate encoding and reversible cross-linking, provides a promising approach for developing dynamic hydrogels that can be finely tuned at the molecular level [28,29]. DNA-based hydrogels provide precise control over stress relaxation, cross-linking thermodynamics, kinetics, and degradability, making them highly adaptable for use in tissue engineering [30,31]. These programmable features allow DNA hydrogels to respond to physical, biological, and chemical stimuli, thereby creating a dynamic extracellular environment conducive to cellular growth, proliferation, and differentiation [[32], [33], [34], [35]].

The use of DNA hydrogels in tissue engineering is still in its early stages, but their potential is already being recognized. DNA-encoded hydrogels can mimic the viscoelastic properties of cartilage ECM, offering an innovative platform for 3D bioprinting and the construction of cartilage organoids. Moreover, the development of L-DNA hydrogels has addressed some of the key challenges associated with D-DNA hydrogels, such as rapid degradation and immune response, by enhancing their stability and anti-inflammatory properties [36]. Recent research has demonstrated the potential of DNA hydrogels in supporting the growth, proliferation, and differentiation of mesenchymal stem cells (MSCs) within a 3D culture environment [37]. For instance, aptamer-DNA hydrogels have been developed to specifically recognize and capture MSCs, thereby improving their retention and differentiation at cartilage injury sites [38]. Additionally, the integration of DNA hydrogels with other materials, such as polylysine or silk fibroin, has led to the creation of hybrid hydrogels with enhanced mechanical properties and resistance to enzymatic degradation [6,39]. Notably, DNA-incorporated SF microspheres have demonstrated the capacity to serve as precursors for the development of cartilage organoids, effectively laying the foundation for more advanced tissue constructs [40].

**Fig. 1:**
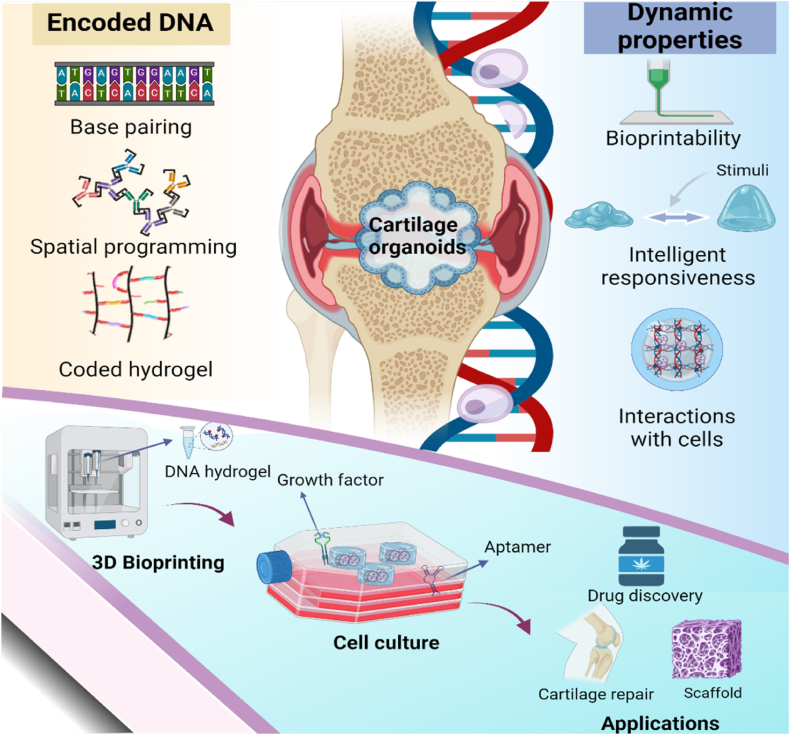
Schematic illustration of the DNA-encoded bioink for constructing cartilage organoids. Created with BioRender.com.

These advances reveal the potential of DNA-encoded bioinks for developing cartilage organoids. Herein, we reviewed the design and fabrication principle for DNA-encoded hydrogels, as well as the predictable dynamic mechanical properties for 3D printing. With special emphasis, this review describes the specific interactions of DNA-encoded hydrogels with cells behavior, and implications for the construction of cartilage organoids (Fig. 1). Although DNA-encoded hydrogels are in their infancy, this review article aims to provide directions for guiding insightful research studies in the construction of cartilage organoids.

## 2 Dynamic hydrogels for cartilage organoids

### 2.1 Dynamics of cartilage tissue

Articular cartilage possesses unique functional properties that derive primarily from its intricate ECM structure and dynamic, viscoelastic characteristics, which enable it to effectively distribute mechanical loads during joint movement [41]. Structurally, articular cartilage can be divided into five zones, each with distinct mechanical characteristics: superficial zone, middle zone, deep zone, calcified zone, and subchondral bone. In the superficial zone, collagen fibers are aligned parallel to the joint surface, effectively dispersing tensile stress induced by movement or external forces, thereby preventing surface cracks and damage. Additionally, the superficial cartilage effectively resists shear stress, reducing friction during movement and maintaining joint surface stability [42]. The middle zone, characterized by a higher density of collagen fibers, can withstand and distribute pressure from the joint. Unlike the superficial zone, the collagen fibers in the middle zone are not aligned parallel to the joint surface, resulting in relatively lower tensile strength [43]. The deep zone has a dense matrix and vertically aligned collagen fibers, enabling it to withstand and distribute compressive forces from joint loads [44]. However, its ability to distribute shear forces is relatively limited [45]. The calcified zone, characterized by its high deposition of calcium salts, can withstand joint load pressures and effectively transmit these pressures to the bone tissue. However, due to the rigidity of its matrix and calcium salt deposition, the calcified zone has low tensile strength and is not suitable for bearing tensile forces [46]. The subchondral bone, composed of high-density bone matrix and trabeculae, is rich in calcium salts and organic components. Its high compressive strength and elasticity effectively bear and distribute joint loads, enhancing structural stability.

**Fig. 2:**
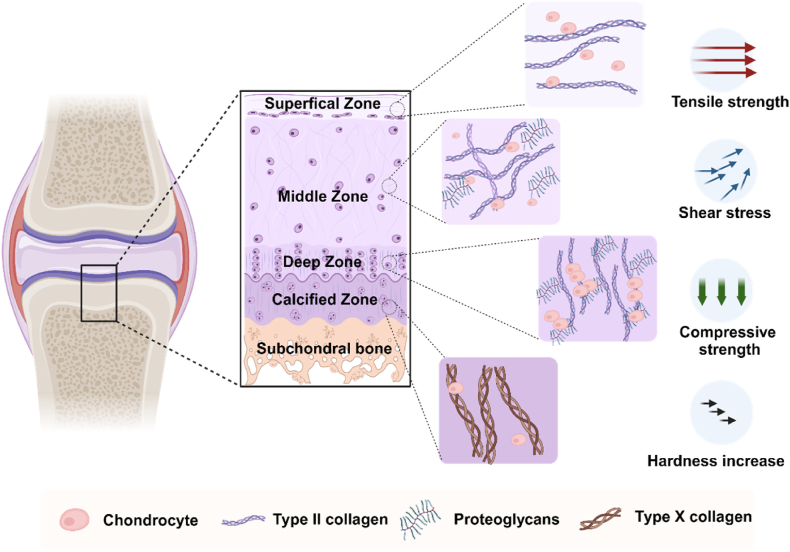
The characteristics and mechanical properties of natural cartilage. Created with BioRender.com.

Notably, the dynamic nature of articular cartilage has a significant impact on cell behavior. Stem cells interact with the ECM's dynamic network through mechanoreceptors, sensing mechanical signals that regulate biological functions such as proliferation, differentiation, and ECM synthesis [47]. This mechanotransduction enhances cellular adaptability by reorganizing the cytoskeleton. The responsiveness of the ECM to mechanical stimuli—facilitated by reversible cross-linking and supramolecular network adjustments—promotes chondrocytes to increase the synthesis of proteoglycans and collagen under compression, thereby supporting cartilage regeneration (Fig. 2).

### 2.2 Dynamic hydrogels for cartilage organoids derivation

**Fig. 3:**
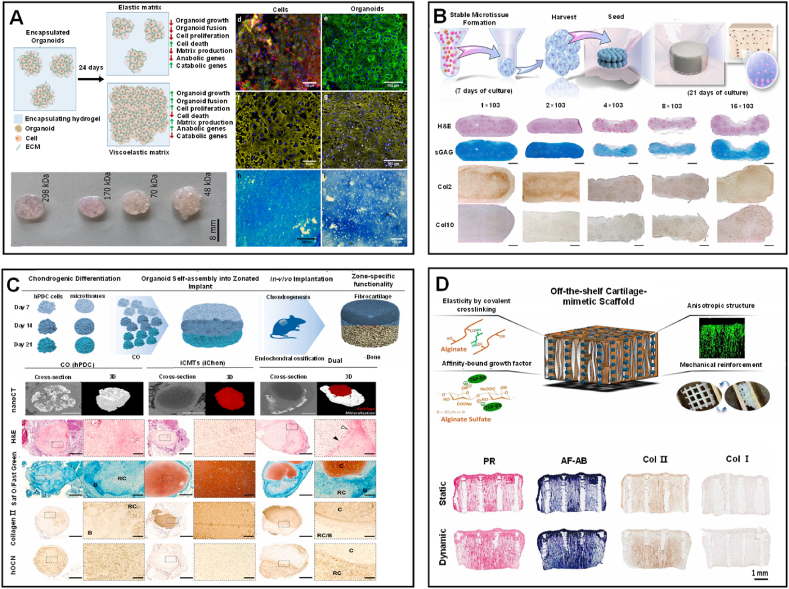
(A) Schematic illustration of viscoelastic hydrogels for the growth and fusion of cartilage organoids. Reprinted with permission from Ref. [57]. Copyright 2021, Elsevier. (B) Pathway of cartilage formation through the self-organization of early cartilage microtissues and histological images of large cartilage tissues. Reprinted with permission from Ref. [18]. Copyright 2022, Elsevier. (C) Schematic illustration of assembling building blocks into a compartmentalized structure, along with cross-sectional and 3D rendered images of mineralized nano-OCT. Reprinted with permission from Ref. [49]. Copyright 2021, Elsevier (D) Schematic illustration of MSCs implanted in a composite scaffold and cultured for in vitro cartilage formation in a dynamic cultivation system. Reprinted with permission from Ref. [52]. Copyright 2022, IOP Publishing Ltd.

The development of cartilage organoids relies heavily on the properties of the scaffold or matrix materials used, which play a crucial role in promoting cell differentiation and tissue maturation. Various studies have explored the use of diverse matrix materials, including hydrogels, ECM-derived inks, and synthetic polymers, to create supportive environments for cartilage tissue engineering. For instance, viscoelastic hydrogels have demonstrated superior ability to support the growth and fusion of cartilage organoids compared to elastic hydrogels, which tend to restrict tissue expansion and integration [[48], [49], [50]] (Fig. 3A). These viscoelastic hydrogels, composed of materials such as alginate or decellularized ECM, provide a mechanical environment that allows cells to proliferate and differentiate into cartilage tissue, producing neohyaline-cartilage with properties resembling native cartilage. Another approach involves using developmentally inspired tissue engineering strategies, such as spatial patterning of phenotypically distinct microtissues within polymeric frameworks, which facilitates the formation of structurally organized cartilage and bone-like tissues [50,51] (Fig. 3B). Additionally, tissue-engineered cartilage is constructed using cartilage decellularized extracellular matrix (dECM). The dECM scaffold exhibits natural adhesion to cells and growth factors, while maintaining biocompatibility and biodegradability, and also possesses mechanical and chemical stability [49] (Fig. 3C). Moreover, some decellularized meniscus ECM-derived inks possess rheological properties that provide excellent printability, making it possible to fabricate cartilage tissue equivalents with mechanical functionality [52] (Fig. 3D).

Additionally, cell phenotype are also crucial aspects in the construction of cartilage organoids [51,53]. Recently, Huo et al. reported that cell adhesion proteins play a key role in regulating cell aggregation and cytoskeletal remodeling during cartilage organoid formation, which not only maintains the cartilage phenotype but also promotes the deposition of cartilage-specific ECM, essential for mimicking the natural cartilage environment [54]. Another study proposed an innovative approach to assemble human chondrocytes into cartilage organoids, utilizing a notochord-derived matrix. The resulting organoids exhibited significant similarity to OA and normal differentiated (ND) chondrocytes, with comparable proliferation rates and ECM production [55]. Guilak et al. induced pluripotent stem cells (iPSCs) into chondrocyte and osteoblast lineages, effectively simulating the process of endochondral ossification, and the resulting organoids contained both cartilage and calcified bone regions [56].

**Fig. 4:**
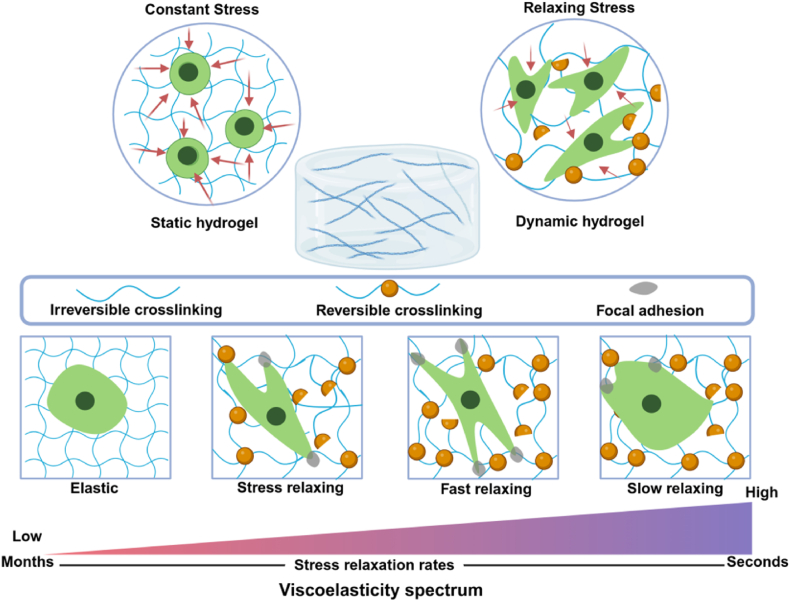
Schematic illustration of dynamic hydrogels interaction with cells. Created with BioRender.com.

Despite these advances, a significant limitation of the current matrix materials used in cartilage organoid development is their limited responsiveness and dynamic interaction with the surrounding environment. Dynamic hydrogels, which can adapt their mechanical properties in response to external stimuli, are increasingly being recognized as a potential solution to this limitation. Dynamic hydrogels are characterized by reversible chemical linkages that allow the network to adapt in response to cellular activities, closely mimicking the dynamic nature of the native ECM [58,59]. These hydrogels provide a tunable mechanical environment that facilitates matrix remodeling, stress relaxation, and other dynamic properties essential for effective 3D cell culture and tissue morphogenesis, thereby making them particularly suitable for organoid construction [59,60]. Moreover, dynamic hydrogels enable controlled degradability, which supports the morphogenetic events necessary for organoid maturation. For example, the incorporation of matrix metalloproteinase (MMP)-cleavable sequences into hydrogels allows local degradation by cell-secreted MMPs [59,61]. This controlled degradation mimics the natural ECM remodeling during organ development, promoting better structural organization and maturation of organoids (Fig. 4).

Although dynamic hydrogels provide a more supportive environment for 3D cell culture, a major challenge in the construction of organoids lies in the precise fabrication of these hydrogels with predictable and tunable properties, such as stiffness, stimulus responsiveness, and stress relaxation. Achieving a controlled and reproducible modulation of these properties is crucial for ensuring the optimal mechanical and biochemical environment that can guide organoid development effectively, yet remains a prominent hurdle in advancing this technology.

## 3 DNA-encoded dynamic hydrogels

The specific complementary pairing rules of DNA molecules (i.e., base pairing between A and T, and C and G) lead to the formation of stable non-covalent interactions. DNA-mediated supramolecular cross-linking utilizes the hydrogen bonding interactions between these complementary base pairs to precisely assemble different molecules or polymer chains into an ordered network structure. The advantage of this approach lies in the highly specific interactions provided by DNA pairing, which allow for precise molecular recognition and assembly. The inherent properties of DNA-encoded sequences and their highly precise structural assembly offer a solid foundation for manipulating both the microscopic and macroscopic properties of DNA-based hydrogels, which enables DNA hydrogels to better accommodate the needs of different cell types and provide a dynamic mechanical microenvironment similar to that of native tissues [62]. For instance, the stress-relaxation properties of DNA hydrogels facilitate cell spreading and invasion in 3D culture, enhance receptor expression, and improve endocytosis at the cell membrane, thereby significantly increasing cell migration and tissue formation capacities within organoids [63]. Furthermore, DNA matrices exhibit excellent self-healing, printability, and stability, and can achieve precise control over mechanical properties by regulating their cross-linking density—features that are crucial for the long-term culture and maturation of organoids [13].

### 3.1 Editability in molecular and spatial levels

**Fig. 5:**
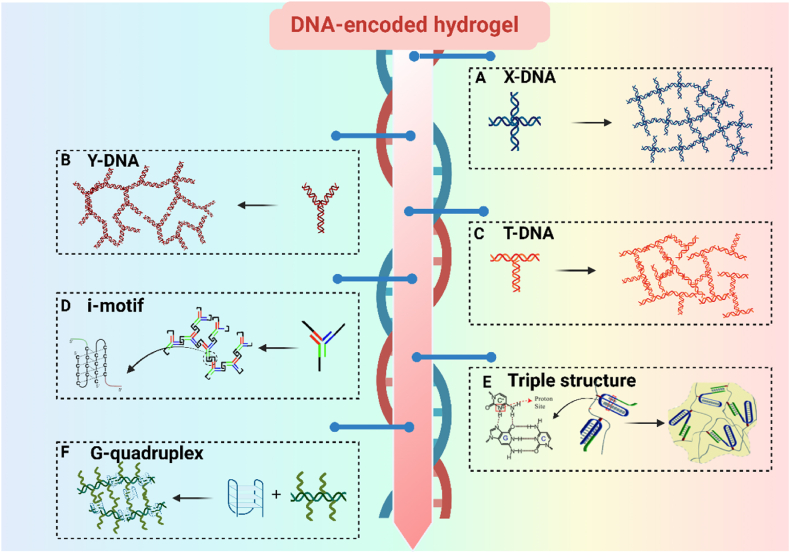
DNA-encoded hydrogel (A) Synthesis of DNA hydrogels using X-DNA. (B) Synthesis of DNA hydrogels using Y-DNA. (C) Synthesis of DNA hydrogels using T-DNA. (D) Synthesis of DNA hydrogels using i-motif [76]. (E) Synthesis of DNA hydrogels using triple structure [77]. (F) Synthesis of DNA hydrogels using G-quadruplex. Created with BioRender.com.

Specific DNA sequences can self-assemble into various spatial structures, including X-shaped, Y-shaped, T-shaped, i-motif, triplex, and quadruplex configurations (Fig. 5). These sequences can be connected to form a 3D network, enabling spatial structural editability [64,65]. For example, X-shaped, Y-shaped, and T-shaped DNA are branched DNAs with complementary sticky ends that can be catalytically ligated by T4 DNA ligase (Fig. 5A, B, C). The porosity and mechanical properties of the resulting DNA hydrogels can vary with the initial concentration and type of the branched DNA [66].

In recent years, some significant non-double-stranded supramolecular DNA insertions, such as i-motif structures [67], triple structures [68,69], and G-quadruplexes [70,71], have further expanded the geometric configurations of DNA polymer chains. The i-motif DNA structure is a particular four-stranded DNA structure that typically forms in DNA sequences rich in cytosine (C) [28] (Fig. 5D). This structure is formed by two parallel cytosine-rich strands that pair via cytosine-cytosine base pairs (C·C+) complementarity. The formation of the i-motif structure is pH-dependent, allowing for rapid switching between gel and non-gel states in response to changes in pH. Therefore, it is more likely to form under acidic conditions and may dissociate under neutral or alkaline conditions [72].

Triple-stranded DNA structures typically involve three strands, where one strand forms Hoogsteen or reverse Hoogsteen pairing with the main strand of the double-stranded DNA, thereby creating a triple helical structure [68] (Fig. 5E). The stability of this structure is influenced by various factors, such as the number and nature of the triplex bridges, mutations in the triplex structural domains, environmental pH, and the presence of ions or ligands in the solution [73].

The G-quadruplex structure of DNA is formed by sequences rich in guanine (G) through specific stacking arrangements [74,75]. In this arrangement, four G bases form a planar tetramer, known as a G-quartet, through Hoogsteen pairing. Multiple G-quartets can then stack vertically via π-π stacking interactions, resulting in a stable four-stranded structure [75] (Fig. 5F). Liu et al. created peptide-DNA conjugates and G-quadruplexes to form supramolecular hydrogels with thermal and ion-responsive properties. These hydrogels transition between gel and sol states based on temperature and potassium ion concentration. Below the thermal transition temperature, the hydrogel is a gel, while above it, the hydrogel becomes a sol.

### 3.2 Gelation strategies of DNA hydrogels

DNA hydrogels can be formed through various crosslinking strategies, primarily including physical crosslinking and chemical crosslinking. Physical crosslinking employs non-covalent bonds, including host-guest interactions, hydrogen bonding, hydrophobic interactions, and metal ion coordination [78]. These bonds can provide dynamic and adjustable properties [79,80]. Conversely, chemical crosslinking, through intermolecular covalent bonds within DNA strands, provides stable and irreversible structures, often resulting in enhanced mechanical strength and durability [78]. In developing dynamically adjustable DNA hydrogels, gelation strategies are broadly categorized into DNA sticky end-induced gelation, enzyme-induced gelation, and hybrid chain reaction-based gelation, each offering distinct pathways to modulate gel properties effectively. Additionally, DNA hydrogels can also cross-link with different polymers to form multi-network hydrogels.

#### 3.2.1 DNA sticky end-induced gelation

**Fig. 6:**
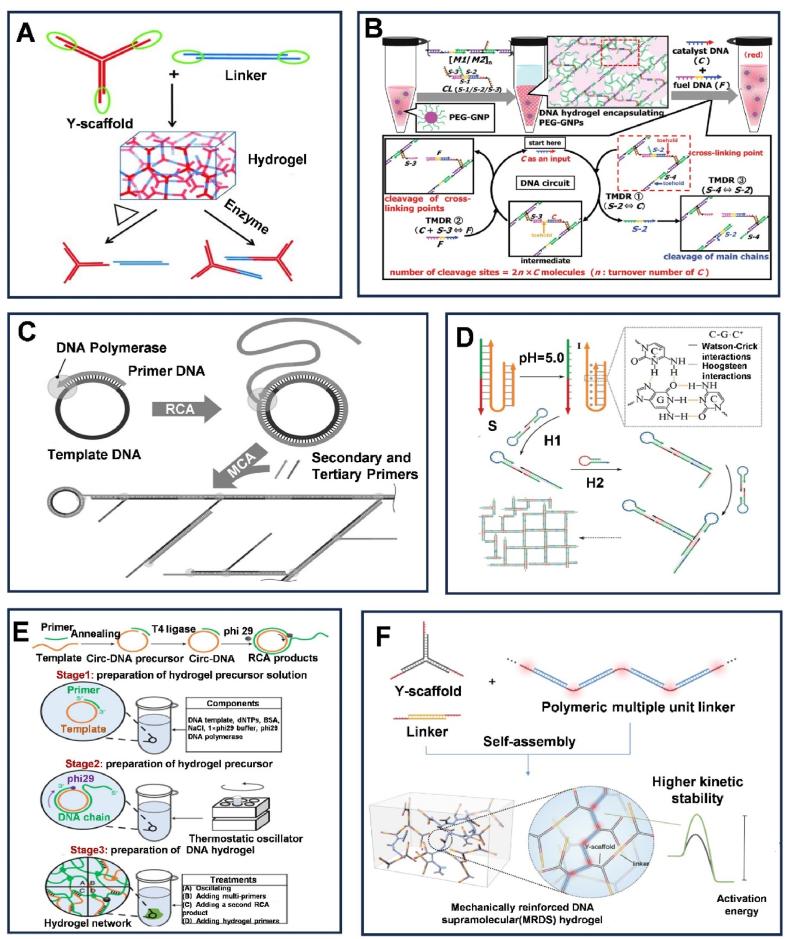
Crosslinking of DNA hydrogels. (A) Schematic illustration of DNA building blocks (Y scaffolds and junctions) self-assembling into DNA hydrogels through the hybridization of their 'sticky ends'. Reprinted with permission from Ref. [82]. Copyright 2010, Wiley. (B) Schematic illustration of the synthesis of dynamically programmed DNA hydrogels mediated by cascade toehold-mediated DNA displacement reactions (TMDR). Reprinted with permission from Ref. [83]. Copyright 2019, Wiley. (C) Schematic of enzyme-based physical DNA hydrogel preparation. Reprinted with permission from Ref. [85]. Copyright 2020, The Society of Polymer Science. (D) Schematic illustration of pH-responsive DNA hydrogel synthesis based on HCR. Reprinted with permission from Ref. [90]. Copyright 2019, The Editorial Department of Chemical Research in Chinese Universities and Springer-Verlag GmbH. (E) Schematic illustration of RCA-based DNA hydrogel synthesis. Reprinted with permission from Ref. [91]. Copyright 2021, Nature Protocols. (F) Schematic illustration of PUML crosslinked with DNA molecules to form mechanically enhanced MRDS hydrogels. Reprinted with permission from Ref. [96]. Copyright 2022, Chinese Chemical Society.

Sticky ends are single-stranded (ssDNA) overhangs of a DNA molecule that extend beyond their complementary strands in the double helix, and they are widely used for the fabrication of DNA hydrogels [81]. For instance, Xing's team developed a DNA hydrogel using complementary sticky ends of two DNA constructs: Y-DNA and L-DNA. Y-DNA, formed from three single strands, has three arms with sticky ends [82]. These ends pair with the complementary sticky ends of L-DNA. By combining Y-DNA and L-DNA in the correct proportions, they formed a hydrogel. The hydrogel's thermal responsiveness depends on the length and composition of the sticky ends (Fig. 6A). Additionally, various functional sequences can be precisely integrated into the hydrogel through sticky end mediation. Oishi et al. developed a special DNA-responsive DNA hydrogel through meticulously designed DNA circuits [83]. This hydrogel utilizes short linear double-stranded DNA (dsDNA) with sticky ends to crosslink the main chain of long linear dsDNA in the hydrogel, which can achieve a sol-gel transition based on the addition of different DNA strands (Fig. 6B).

#### 3.2.2 Enzyme-induced gelation

Enzymes are key molecular agents that facilitate the assembly of DNA hydrogels, particularly polymerases and ligases [66]. Traditionally, the synthesis of DNA hydrogels requires sufficiently long sticky ends to ensure the stability of the cross-linkers [81]. Ligase, however, is an enzyme that connects DNA fragments with complementary sticky ends and repairs dsDNA gaps, promoting covalent cross-linking of these ends to form hydrogels. For instance, the use of T4 DNA ligase has enabled the hybridization and connection of branched DNA molecules, which serve both as monomers and crosslinkers, leading to the formation of large-scale 3D hydrogel structures [66]. Moreover, linear dsDNA obtained from plasmid DNA through enzymatic digestion is also an ideal DNA building block for DNA hydrogels. For instance, the plasmid pUC19 is cut by two different nucleases, producing two linear dsDNA fragments with sticky ends that include self-complementary palindromic sequences. These fragments are then separated and, with the help of ligase, subsequently form a DNA hydrogel [84]. Additionally, Hamada's team developed DNA materials with artificial metabolism based on physical DNA hydrogel technology [85] (Fig. 6C). These materials are synthesized by Phi29 DNA polymerase from nanoscale building blocks (dNTPs) to form micrometer-scale DNA chains, akin polymerase chain reaction (PCR) to the original physical DNA hydrogel. Meanwhile, Hartman et al. used Taq polymerase and PCR [86] to create heat-resistant DNA hydrogels. They formed Y-shaped DNA from three single strands, which self-assembled into branched structures [87,88]. Psoralen treatment linked these structures into dumbbell shapes. These linked Y-DNA structures served as templates for further PCR, highlighting the role of polymerase in creating durable DNA hydrogels. In addition, terminal deoxynucleotidyl transferase (TdT) can be used to create DNA hydrogels by adding nucleotides to the ends of DNA strands without a template [89].

#### 3.2.3 Hybrid chain reaction-based gelation

Hybridization chain reaction (HCR) is a unique isothermal DNA strand displacement process involving two hairpin structures. It facilitates the formation of cross-opening polymer nucleic acid chains, serving as a universal strategy to fabricate reactive hydrogels with tunable properties [90]. For instance, Li et al. developed a pH-triggered DNA hydrogel using clamp hybridization chain reaction (C-HCR) [90]. At pH 5.0, a special nucleic acid switches to a C-G·C^+^ triplex, releasing an initiator molecule. This initiates a chain reaction, forming a multi-armed structure. Combining two structures, H1 and H2, creates a stable 3D DNA hydrogel, suitable for drug delivery, biosensing, and tissue engineering (Fig. 6D). Rolling circle amplification (RCA) is another efficient strategy for the synthesis of DNA hydrogels, producing long DNA chains from a circular template under isothermal conditions, suitable for forming physically cross-linked hydrogels. Yao and team created DNA hydrogels using RCA to form a network of ultra-long DNA strands [91] (Fig. 6E). This hydrogel flows like a liquid outside of water but remains solid in water. They further developed a physically crosslinked RCA-based hydrogel for capturing, 3D encapsulating, and enzyme-triggered release of bone marrow mesenchymal stem cells (BMSCs) [92].

#### 3.2.4 Hybrid DNA hydrogels

Although DNA hydrogels possess many attractive characteristics, they also have certain disadvantages. For instance, unmodified DNA hydrogels tend to be soft, which limits their practical applications due to the difficulty in adjusting the stiffness of the hydrogels [93]. Significantly, hydrogels that engage in DNA hybridization have garnered widespread interest. The incorporation of a secondary crosslinking network into the hydrogel structure allows for the amalgamation of the physicochemical properties of the two distinct networks. This interpenetration of networks facilitates the enhancement or conveyance of particular attributes, such as mechanical strength [94]. Furthermore, by modulating the viscosity of the continuous aqueous phase, the incorporated polymer can significantly improve the hydrogels' stability [95]. Meanwhile, the hybridized DNA hydrogels retain the editability of DNA, showing great potential for biomedical applications. Lachance‐Brais developed an injectable DNA-based hydrogel to increase the stiffness of DNA hydrogels [93]. The hydrogel forms multi-micron supramolecular fibers through the interaction between polyadenine (dA) and cyanuric acid (CA). By altering the composition, the mechanical properties of the hydrogel can be adjusted. Li et al. showcased a strategy for mechanical reinforcement by incorporating a polymer multi-unit linker (PMUL) into a model system of DNA supramolecular hydrogel [96], which is constructed from self-assembling DNA Y-scaffold and linker (Fig. 6F). This approach led to the formation of a mechanically reinforced DNA supramolecular (MRDS) hydrogel. By leveraging multi-unit interactions in the DNA supramolecular hydrogel model system, the linker consolidates various supramolecular units into the polymer main chain, effectuating the crosslinking of the supramolecular hydrogel. This process notably amplifies the mechanical strength of DNA supramolecular crosslinks and augments the stability of the system's dynamics. At the same time, the dynamic properties of the supramolecular hydrogel, such as shear thinning and self-healing, are effectively retained.

## 4 Properties of DNA-encoded hydrogels

### 4.1 Dynamics of DNA-encoded hydrogels

**Fig. 7:**
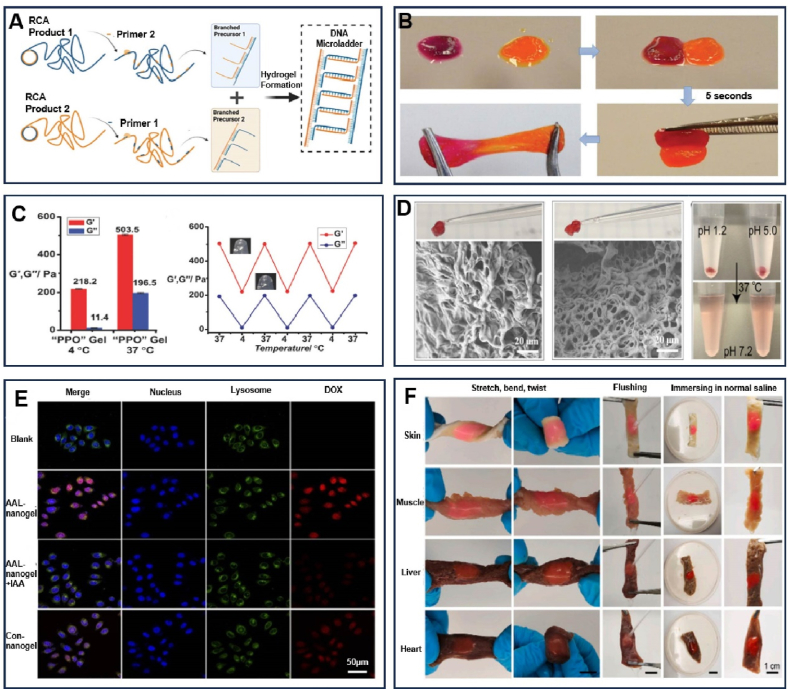
Properties of DNA hydrogels. (A) The formation of DNA micro-ladders. Reprinted with permission from Ref. [99]. Copyright 2023, APL Bioengineering. (B) The rapid self-healing behavior of DNA organic hydrogel. Reprinted with permission from Ref. [98]. Copyright 2021, Wiley. (C) Schematic illustration of the changes in mechanical properties of PPO gels at 4 °C and 37 °C. Reprinted with permission from Ref. [102]. Copyright 2018, Wiley. (D) Photos and SEM images of DNA hydrogels obtained at pH 1.2 and pH 5.0, as well as a schematic illustration of the changes in the morphology of DNA hydrogels at different pH levels. Reprinted with permission from Ref. [108]. Copyright 2022, American Chemical Society. (E) The release of DOX under different samples and conditions. Reprinted with permission from Ref. [109]. Copyright 2022, American Chemical Society. (F) Schematic illustration of the good adhesiveness and ability to withstand curling of the DGL gel. Reprinted with permission from Ref. [110]. Copyright 2023, Elsevier Ltd.

Ordinary hydrogel networks, due to their generally isotropic internal structure, often do not exhibit anisotropic optical, electrical, magnetic, or mechanical properties, limiting the use of dynamic functions within them [97]. Consequently, these limitations can restrict their applications in critical fields. In contrast, DNA hydrogels are known for their dynamic and tunable mechanical properties, such as elasticity and viscoelasticity. This makes them ideal for designing cellular microenvironments that can guide cell behavior and fate. DNA hydrogels exhibit dynamic and adaptive characteristics and have capable of presenting bioactive molecules in a dynamic and reversible manner, allowing control over their cellular interaction [98]. For instance, Hanif et al. based on RCA and multiple primer chain amplification methods, developed a physically crosslinked all-DNA hydrogel, which possesses tunable morphology and controllable biodegradation [99]. Furthermore, they also designed a new DNA micro-ladder structure as a framework for modulating the properties of the hydrogel, and demonstrated how to adjust the characteristics of this DNA-based biomaterial by regulating the number of rigid dsDNA chains (Fig. 7A). Wu et al. created a hydrogel crosslinked with peptides derived from human serum albumin (HSA) and multi-arm DNA, which allows for the convenient immobilization of proteins via ssDNA hybridization [100]. The stiffness of this hydrogel is widely tunable and it also possesses high biocompatibility and enzyme-mediated degradability, making it an attractive candidate as an ECM for 3D cell culture. Li and colleagues developed a supramolecular peptide-DNA hydrogel that crosslinks rapidly and utilized it for precision in-situ multi-layer 3D bioprinting [101]. The standout feature of this hydrogel is its exceptional mechanical strength, coupled with its ability to resist swelling or shrinking. This combination ensures that the bioprinted structures remain geometrically consistent and stable at the millimeter scale, maintaining their integrity without collapsing post-printing. Meng et al. reported a DNA organic hydrogel [98], which can induce mesophase order through supramolecular exchange and complete self-repair within 3 s. More importantly, the DNA-based liquid crystalline organic hydrogels exhibit exceptional ultimate tensile strength exceeding 1 MPa, stiffness over 20 MPa, and toughness up to 18 MJ/m^−3^, indicates the exceptional mechanical properties of DNA-based liquid crystalline organic hydrogels (Fig. 7B). Wu and his team developed a DNA hydrogel with reversible mechanical strength by incorporating polypropylene oxide (PPO) as a thermoresponsive unit into DNA network [102]. Due to the interaction between the DNA network and PPO units, the mechanical strength of the DNA hydrogel can reversibly fluctuate between 218.2 and 503.5 Pa as the temperature shifts from 37 °C to 4 °C (Fig. 7C).

The stimulus responsiveness of DNA hydrogels has garnered significant attention in tissue engineering because they can change their structure in response to one or more stimuli, such as undergoing a significant phase transition from gel to sol, thereby changing their physical and chemical properties [[103], [104], [105]]. Research has found that DNA hydrogels respond to various stimuli, including non-biological factors such as light, temperature, pH, metal ions, as well as biological factors like nucleic acids, enzymes, adenosine triphosphate (ATP), and more [106]. Furthermore, to enable this responsiveness, various functional motifs, including i-motifs, aptamers, and G-quadruplexes, can be integrated into the polymer network for sophisticated molecular recognition, facilitating complex stimulus responses. Lyu et al. discovered a DNA hydrogel [107], which exhibits stimulus-responsive sol-gel transitions due to the opening of DNA sequence motifs, catalyzed by *Eco*R I restriction endonuclease or temperature changes. This phenomenon facilitates controlled release of liposomes. Additionally, this hydrogel also possesses injectability and self-healing capabilities, making it further applicable for other targeted controlled release systems. Hu's team has constructed acid-resistant DNA hydrogels containing an A-motif (parallel double-stranded at pH 1.2–3.0) and a i-motif (G-quadruplex at pH 4.0–6.0) [108]. These hydrogels can effectively pass through gastric juice, duodenal fluid, and intestinal fluid, providing a novel carrier for oral insulin delivery (Fig. 7D). Xu et al. created an ATP-responsive nanohydrogel using polyacrylamide copolymers grafted with DNA. ATP aptamers were linked to this scaffold, and doxorubicin (DOX) was integrated into the nanohydrogel. When ATP is present, the nanohydrogel forms aptamer/ATP complexes, causing the nanogel to disassemble and release DOX for targeted fluorescence imaging and chemotherapy in cancer cells [109] (Fig. 7E).

DNA molecules confer programmability to hydrogels, enabling the design of modified DNA hydrogels with tailored properties for specific biomedical applications. These hydrogels excel in minimizing unspecific protein adsorption while facilitating cell and tissue adhesion, making them ideal for rapid hemostasis and promoting seamless wound healing. Xie and his team designed a nanoscale DNA supramolecular hydrogel sealant, referred to as DGL [110], by incorporating polymerizable gelatin methacrylate (GelMA) and Laponite nanoclay, which have anisotropic surface charges, into the DNA matrix. This innovation leads to a versatile physical/chemical crosslinking network that boasts adjustable dynamic mechanical properties and robust tissue adhesion for efficient hemostasis (Fig. 7F). Drawing inspiration from mussel adhesives and the inherent properties of DNA, Ma and colleagues crafted a conductive, self-healing hydrogel (CaSA-GAD hydrogel) with a triple-cross-linking network involving calcium ions (Ca^2+^) [111]. It demonstrates peelable properties in tissue adhesion, and also features high toughness and self-healing abilities, further ensuring the mechanical performance of the hydrogel.

### 4.2 Modulation of DNA-encoded hydrogels

**Fig. 8:**
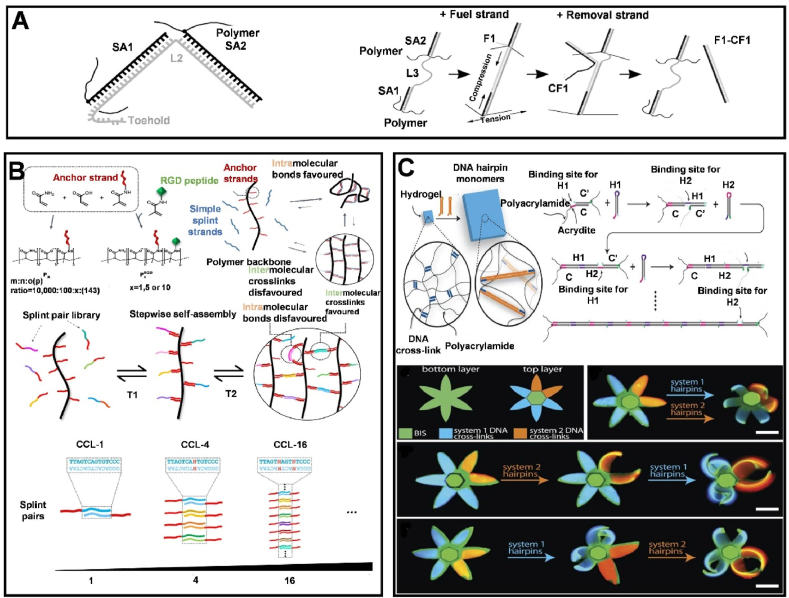
Regulation of the cross-linking effect of DNA hydrogels (A) Adjusting the mechanical properties of DNA hydrogels according to the length of the cross-linker. Reprinted with permission from Ref. [114]. Copyright 2005, The Materials Research Society. (B) The basic concept and cross-linker design of DyNAtrix, as well as the tuning of viscoelasticity according to the number of cross-linkers. Reprinted with permission from Ref. [13]. Copyright 2023, Nature. (C) Regulation of the swelling degree of DNA hydrogels based on the relative concentration of two types of hairpin concentrations. Reprinted with permission from Ref. [115]. Copyright 2023, Science.

To more precisely control the mechanical properties of DNA hydrogel networks, the length and density of DNA crosslinkers are typically adjusted, thereby altering the macroscopic mechanical characteristics of the hydrogels [112]. For example, Previtera et al. synthesized DNA-crosslinked polyacrylamide hydrogels with varying crosslinking lengths, and the hydrogels with longer side chains exhibited significantly higher mechanical strength and stress [113]. After that, Lin et al. [114] also explored the mechanical properties of polyacrylamide gels cross-linked with DNA, creating a hydrogel with controllable mechanical traits. By varying the incorporation of DNA strands, they could adjust the hydrogel's stiffness. Additionally, their hydrogel includes specific 20-mer side branches and a 50-mer oligonucleotide cross-linker, allowing for controlled cross-link dissociation via competitive binding initiated at a designed "toehold" (Fig. 8A).

Recently, Peng et al. have developed fully synthetic hydrogels using DNA libraries, capable of self-assembling with ultrahigh-molecular-weight polymers to create a dynamic, DNA-crosslinked network named DyNAtrix [13]. In this system, polymers attached to DNA anchor chains form a dynamic network through binding with DNA crosslinkers, which consist of two segments, known as splints. By modifying the DNA sequences in the overlapping regions of these splints, DyNAtrix can adapt its viscoelastic, thermodynamic, and kinetic characteristics in a predictable manner. This adaptability allows for the tuning of properties such as stress relaxation time, ranging from under a second to several hours, by selecting different lengths for the overlapping domains (Fig. 8B). Its design, which relies on reversible non-covalent bonds among DNA cross-linkers, endows it with self-healing capabilities. More importantly, the hydrogel has been used for culturing human induced pluripotent stem cells (hiPSCs), supporting their proliferation and differentiation. Cangialosi et al. utilized the 'hairpin structure' of DNA sequences to control the mechanical properties of hybrid hydrogels, such as the swelling and shape changes of the hydrogel [115]. The hairpin structure refers to a formation made up of complementary DNA sequences. In this scenario, the hairpin structures are of two kinds: the 'polymerizing hairpin', which facilitates the expansion of the hydrogel, and the 'terminator hairpin', which serves to limit this expansion. The degree of swelling in the DNA hydrogel can be precisely modulated by fine-tuning the relative concentrations of these two distinct hairpin structures (Fig. 8C).

The concentration of ss DNA fragments can regulate the stiffness of DNA chains [116]. Pan et al. demonstrated this by preparing DNA hydrogels with a combination of flexible, rigid, and mixed chains through a synthesis method involving polymerization followed by cross-linking [116]. Among them, rigid networks utilized two-strand assemblies, while flexible networks were created from a combination of 30-base long ssDNA and 14-base pair long linear dsDNA. Both network types shared a common 10-base pair long dsDNA cross-linking domain. By integrating these networks, a hybrid system with mixed chain properties was achieved, demonstrating that the inclusion of specific proportions of rigid chains could significantly enhance the mechanical strength of hydrogels initially composed of more flexible chains. Furthermore, it allows precise control over the network's structural connectivity and pore size.

### 4.3 Printability of DNA-encoded hydrogels

**Fig. 9:**
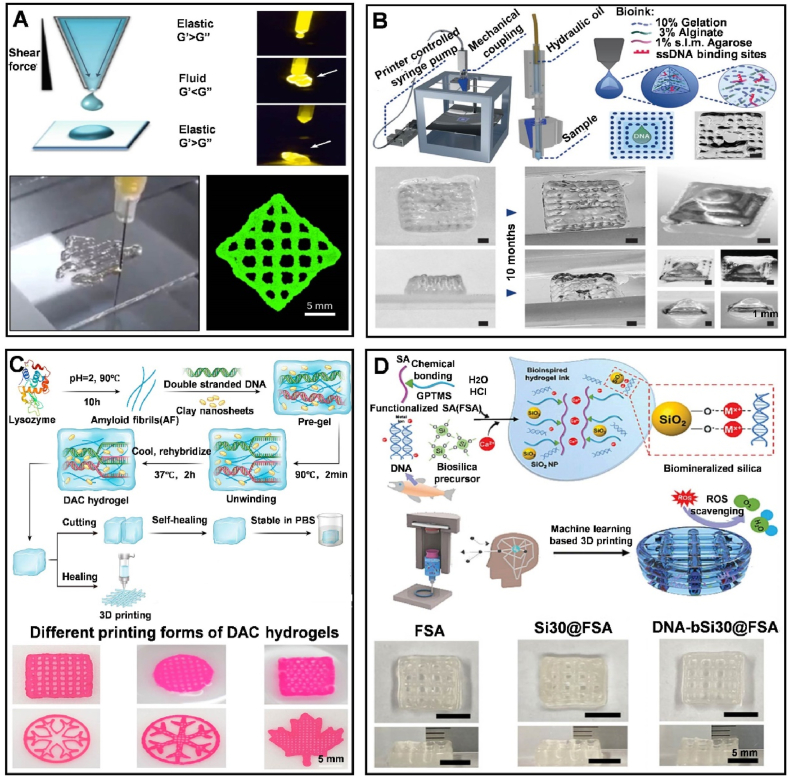
Adjustment of the printability of DNA hydrogels. (A) The printability of DyNAtrix. Reprinted with permission from Ref. [13]. Copyright 2023, Nature. (B) Schematic illustration of hybrid DNA hydrogels composed of gelatin, alginate, and agarose, and their printability. Reprinted with permission from Ref. [123]. Copyright 2020, Wiley. (C) Schematic illustration of the synthesis of dual nanoengineered self-assembling DAC hydrogels and their printability. Reprinted with permission from Ref. [124]. Copyright 2023, American Chemical Society. (D) Schematic illustration of DNA-induced biomineralization for fabricating biomimetic 3D printed hydrogels and their printability. Reprinted with permission from Ref. [125]. Copyright 2023, Wiley.

Utilizing dynamic DNA hydrogels as biological ink for 3D printing cartilage organoids, the printability of DNA hydrogels thus becomes an important property. The printability of hydrogels is closely related to factors such as the viscosity of the hydrogel itself, curing methods (such as photo-crosslinking [117], cryogenic freezing), the swelling rate of the hydrogel [118], the surface tension and wettability of the hydrogel [119] and the temperature during printing. Especially, the viscosity of the hydrogel is crucial in 3D printing as it determines the gel's suitability and quality in extrusion-based bioprinting [120,121]. the viscosity range of hydrogels suitable for extrusion printing is quite broad, varying from 30 mPas to greater than 600 × 10^6 mPas [122]. Peng et al. achieved printability and self-healing in DNA hydrogels by designing complex combinatorial crosslinker libraries (CCLs), incorporating heat-activated crosslinkers (HACs), and utilizing stress-relaxation crosslinkers (SRCs). These strategies allowed precise control over the mechanical properties and thermal response of the hydrogels. The CCLs enhanced crosslinking efficiency, the HACs ensured uniform cell mixing and rapid gelation at 37 °C, and the SRCs enabled tunable stress relaxation. These innovations allowed the hydrogels to remain liquid at low temperatures for easy mixing and printing, and to rapidly solidify post-printing, while maintaining self-healing properties to recover from large deformations [13] (Fig. 9A). Moreover, Müller et al. developed a DNA-functionalized bioink optimized for extrusion-based 3D printing. This bioink, composed of gelatin, alginate, and low-melting agarose, solidifies rapidly at room temperature, ensuring high structural fidelity. The agarose was functionalized with DNA using click chemistry, allowing for sequence-programmable localization and diffusion control within the printed gel. This setup enabled precise deposition and patterning of the hydrogel, suitable for creating complex 3D structures with embedded DNA functionalities [123] (Fig. 9B). Notably, DNA strands can also interact with other materials, such as amyloid fibers and clay nanosheets, through charge adsorption, forming supramolecular hydrogel networks. This interaction not only enhances the mechanical strength and stability of the hydrogels but also imparts excellent properties such as shear thinning, self-healing, and 3D printability [124] (Fig. 9C). Kim et al. designed a bioactive hydrogel ink that combines DNA from salmon sperm with biomineralized silica, incorporated into functionalized alginate. DNA facilitated the biomineralization of silica, allowing the hydrogel to solidify quickly while maintaining structural integrity, making it suitable for extrusion-based 3D printing [125] (Fig. 9D).

In addition, DNA hydrogels can achieve light-cured 3D printing by incorporating light-controlled cross-linking groups such as azobenzene and spiropyran [31,126]. For example, Kandatsu et al. designed a dynamic DNA hydrogel based on photoreversible hybridization. It was synthesized from polyacrylamide combined with azobenzene-modified DNA strands and their complementary strands, and was able to undergo sol-gel phase transitions under ultraviolet irradiation at different wavelengths [127]. Furthermore, Huang and colleagues developed polyacrylamide hydrogels with DNA modules modified by various o-nitrobenzyl phosphate esters, enabling light-induced patterning within the hydrogel matrix to form spatially ordered patterns with programmable functions [128].

## 5 Materiobiological effects of DNA-encoded hydrogels

The intricate interactions between cells and the ECM depend on the temporal dynamics and spatial definition of physical and chemical cues. DNA hydrogels possess customizable dynamic structures that can mimic the natural ECM, guiding appropriate cell phenotypes. Compared to traditional static hydrogels, dynamic DNA hydrogels not only meet the biological and mechanical requirements of bioinks but also respond in situ to cellular outputs or external stimuli. These characteristics bring hope for the construction of cartilage organoids.

### 5.1 Cellular interactions with DNA-encoded hydrogels

**Fig. 10:**
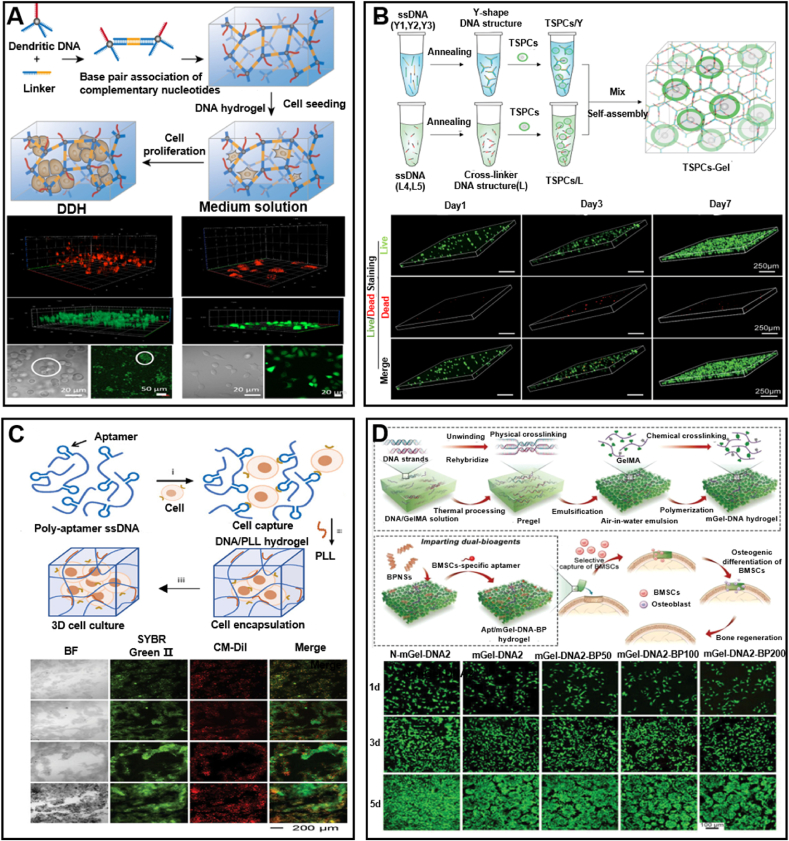
Culturing cells in DNA hydrogels. (A) Synthesis of dendritic DNA hydrogels and schematic illustration of cell viability within them. Reprinted with permission from Ref. [131]. Copyright 2021, American Chemical Society. (B) Synthesis of TSPC-Gel and schematic illustration of TSPC proliferation and survival in DNA hydrogels. Reprinted with permission from Ref. [132]. Copyright 2023, Wiley. (C) The synthesis of DNA/PLL hydrogel for 3D cell culture and fluorescence microscopy images of the 3D culture of U87 cells. Reprinted with permission from Ref. [134]. Copyright 2024, Wiley. (D) Synthesis of mGel-DNA double network macroporous hydrogels and schematic illustration of BMSCs viability on double network hydrogels. Reprinted with permission from Ref. [135]. Copyright 2023, Wiley.

Specifically, DNA hydrogels can serve as ideal cell carriers, providing appropriate mechanical support and biological protection to prevent cells from being damaged by shear forces or the immune system during transport [129,130]. Wu et al. [131] fabricated the DNA hydrogel by self-assembling dendritic DNA molecules with four branches. This hydrogel demonstrates adjustable mechanical strength and reversible thixotropy even at nanomolar DNA concentrations. It can isothermally convert the cell culture medium into a hydrogel at physiological temperature, allowing in situ seeding and high proliferation of both cancer and somatic cells. The specific loading of bioactive cues is enabled by the bis-entity of dendritic branches, which helps regulate cell behaviors. The advantages of this dendritic DNA-assembled hydrogel include high biocompatibility, easy functionalization, and the ability to form complex cell morphologies such as spheroids. This makes it an effective platform for 3D cell culture applications (Fig. 10A). Moreover, the DNA hydrogel was found to effectively enhance tendon stem/progenitor cells (TSPCs) therapy by maintaining cell viability and function, as well as promoting the healing of Achilles tendinopathy in rat models. This method shows potential for improving tendon repair by providing a suitable microenvironment for TSPCs, leading to better retention and integration of these cells in the tendon tissue [132] (Fig. 10B). In addition, DNA hydrogel with high mechanical strength, thixotropy, and improved biostability can be achived by using of L-DNA, which could be resistant to nuclease degradation, making it suitable for long-term cell culture with minimal inflammatory response.

Notably, DNA-based multi-network hydrogels have also become excellent platforms for cell culture. For example, the dendritic DNA nanostructures was functionalized with photo-cross-linkable and cell adhesion moieties to form DNA hydrogel [133]. PEG was added to provide structural support and enhance the hydrogel network's stability. This incorporation helps maintain structural integrity, allowing better cell encapsulation and enhancing cell viability and distribution within the hydrogel matrix. Tang and colleagues introduced a new type of DNA hydrogel for 3D cell culture [134]. Unlike conventional DNA hydrogels, this hydrogel employs poly-L-lysine (PLL) as both a cross-linker and a protective agent to resist nuclease degradation. Notably, the integration of poly-AS1411 aptamers into the DNA/PLL hydrogel for anchoring U87 cells demonstrated that the cell encapsulation efficiency of the DNA/PLL hydrogel with aptamers was four times higher than that of the hydrogel without aptamers (Fig. 10C). Furthermore, gelatin methacryloyl (GelMA) combined with DNA formed a double-network macroporous hydrogel, in which GelMA provided the structural framework, and DNA enhanced mechanical strength and stability through a physical cross-linking network [135]. Besides, its 3D macroporous structure offers an enhanced spatial environment conducive to the proliferation of stem cells (Fig. 10D).

In addition to the existing research, several innovative approaches involving nano-engineered DNA microbeads have recently attracted considerable attention; for instance, Göpfrich et al. introduced nano-engineered DNA microbeads with tunable stiffness, designed to mimic the mechanical properties of tissues, which were utilized as a modifiable DNA hydrogel material, enabling the precise spatiotemporal control of morphological gradients at any stage of organoid development, thus providing a versatile platform for the manipulation of organoid structures [136]. Remarkably, microinjection of these DNA microbeads does not interfere with the normal development of the organoids, and, once the tissue is integrated through light-triggered disruption, the process allows for non-invasive erasure, showcasing the potential of this system for dynamic manipulation of organoid growth. Moreover, this research underscores the ability of DNA microbeads to enhance the complexity of organoid culture systems and improve the accuracy of their phenotypic representation, thereby advancing the field of tissue engineering.

Although DNA hydrogels are known for their tunable mechanical properties, their intrinsic mechanical strength is somewhat limited; therefore, by constructing a transparent 3D system through the incorporation of a second network covalently crosslinked within the DNA supramolecular hydrogel, researchers have significantly enhanced the mechanical strength of these hydrogels, making them more suitable for applications requiring greater structural integrity. For example, Cao and colleagues introduced AAm/BIS monomers into the DNA hydrogel after cell culture, employing in situ polymerization to form the secondary network, which effectively immobilized cells in a 3D matrix for subsequent immunostaining and imaging [137]. This methodological approach provides valuable insights into the behavior and functionality of cells within a 3D environment, offering significant potential for studying cellular dynamics. To further enhance the mechanical properties of DNA hydrogels, recent work by Dong et al. has focused on developing a strategy to adjust the stiffness of these hydrogels by manipulating the rigidity of the DNA modules used in their construction [138]. By incorporating building blocks with higher molecular stiffness and optimizing their connectivity, this approach allows for the systematic modulation of the stiffness of the hydrogels, thereby offering greater control over their mechanical properties. These advanced hydrogels, with their excellent dynamic performance and biocompatibility, hold considerable promise for use in 3D cell culture systems, particularly in applications that require precise control over the mechanical environment of the cells.

### 5.2 Bioactive compounds incorporated in DNA-encoded hydrogels

Unlike native ECMs, synthetic hydrogel scaffolds lack the traditional patterns that act as "bait" for attracting cells [139]. By incorporating bait molecules (such as adhesion factors and growth factors) into DNA hydrogels, precise interactions between the gel matrices can be achieved, thus providing a more controlled microenvironment, which is crucial for organoid construction [140].

#### 5.2.1 Adhesives

**Fig. 11:**
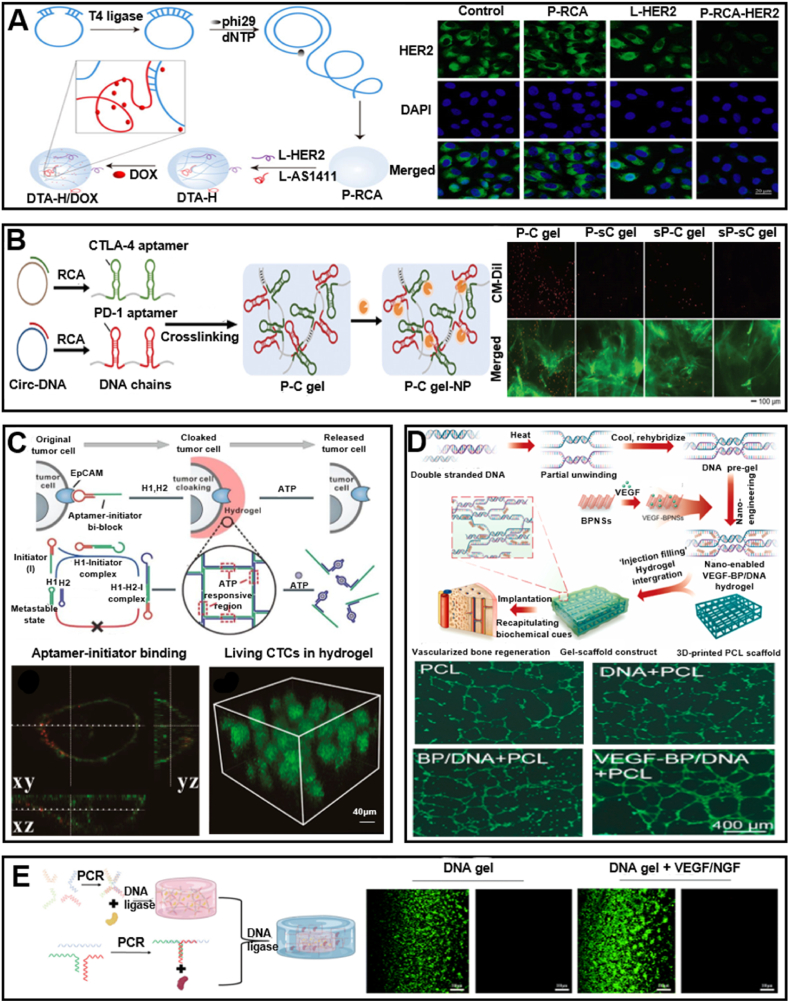
Incorporation of active substances in DNA hydrogels. (A) Synthesis of DTA-H and schematic illustration of the viability of SK-BR-3 cells. Reprinted with permission from Ref. [144]. Copyright 2022, Frontiers. (B) Schematic illustration of the synthesis of P-C gel-NP and the activity of T cells in different hydrogels. Reprinted with permission from Ref. [145]. Copyright 2024, Wiley. (C) Schematic illustration of CTC release in aptamer-functionalized DNA hydrogels and the viability and distribution of cells on them. Reprinted with permission from Ref. [146]. Copyright 2017, American Chemical Society. (D) Schematic illustration of the synthesis of DNA hydrogels supporting BPNS loaded with VEGF, and fluorescence images of HUVECs. Reprinted with permission from Ref. [169]. Copyright 2022, Elsevier. (E) Construction of DNA hydrogels + VEGF/NGF and schematic illustration of RSC cell viability in the hydrogels. Reprinted with permission from Ref. [170]. Copyright 2021, Royal Society of Chemistry.

Adding adhesion factors, such as aptamers and arginine-glycine-aspartic acid (RGD) peptides, to DNA hydrogels can significantly enhance their ability to adhere to tissues or recruit cells. Aptamers are specialized short sequences of DNA or RNA that can adopt secondary and tertiary structures upon encountering specific targets, including molecules on the cell surface. This allows them to bind to cell surface molecules with higher affinity and unique specificity [[141], [142], [143]]. Jin and colleagues designed a dual-targeting aptamer-decorated DNA hydrogel (DTA-H) for effective, consistent, and precise drug delivery. The hydrogel system easily integrates HER2 aptamer and AS1411 aptamer into DTA-H through DNA complementary base pairing. In this context, the multivalent decoration of aptamers enhances the system's binding affinity to target cells, thereby increasing the efficiency of drug delivery [144](Fig. 11A). Furthermore, Zhang and colleagues developed a DNA hydrogel containing polyvalent aptamers targeting PD-1 and CTLA-4, which are key immune checkpoints in T cells [145]. This hydrogel is designed to release these aptamers in response to inflammatory signals within the tumor microenvironment, thus allowing for controlled and localized treatment. Moreover, this hydrogel possesses excellent cultivation capabilities for T cells, significantly enhancing the killing efficiency of T cells against tumor cells (Fig. 11B). Furthermore, Song and colleagues proposed introducing small molecule-responsive aptamers into the C-HCR [146]. Upon introduction of the small molecule, the aptamer undergoes a conformational shift from an unfolded state to a tertiary structure, which triggers the breakdown of the 3D DNA network and the release of the pre-encapsulated circulating tumor cells (CTCs). Significantly, this research underscores the substantial potential of DNA for the non-detrimental capture and release of viable CTCs, presenting potential application prospects in the realm of cancer treatment (Fig. 11C).

RGD peptide, as an important bioactive factor, has significant functions in promoting cell adhesion and proliferation. It can provide critical adhesion motifs from the ECM, thereby enhancing cell adhesion on the DNA hydrogel scaffold [147]. Meanwhile, the interaction between the RGD peptide and the DNA structure can enhance the mechanical properties of the hydrogel, thereby affecting cell migration and proliferation by regulating the stiffness of the DNA hydrogel [148]. Yao et al. designed a DNA-based daisy chain rotaxane nanocomposite (DNA-DCR) hydrogel. In this system, the RGD peptide is covalently cross-linked with the DNA hydrogel and integrated into the DNA-DCR. Consequently, the DNA-DCR, through the sliding mechanism of HEX units along the axis, allows the RGD peptide to move, thereby creating a dynamic environment that simulates the characteristics of the ECM. Notably, the RGD peptide offers specific binding sites for integrins on the surface of human bone marrow mesenchymal stem cells (hMSCs). This significantly enhances the migration and proliferation of hMSCs, thereby strengthening their adhesion and interaction with the hydrogel scaffold [149]. Additionally, the RGD peptide can be grafted onto DNA hydrogel microspheres through photopolymerization. These RGD-modified hydrogel microspheres demonstrate exceptional biocompatibility, significantly enhancing the chondrogenic differentiation of BMSCs, thus facilitating the regeneration and repair of cartilage tissue [40].

#### 5.2.2 Growth factor

Growth factors are essential for tissue development, maintenance of homeostasis, and regeneration [150]. It has been reported that a lot of growth factors such as transforming growth factor-β1 (TGF-β1) [[151], [152], [153], [154], [155], [156]], bone morphogenetic protein type 2 (BMP-2) [157,158], transforming growth factor-β3 (TGF-β3) [[159], [160], [161], [162], [163], [164]], insulin-like growth factor 1 (IGF-1) [165], and platelet-rich plasma (PRP) [[166], [167], [168]] can bind with DNA hydrogels to regulate tissue regeneration. Miao et al. designed a dynamic DNA hydrogel using vascular endothelial growth factor (VEGF)-modified BPNSs nanosheets. VEGF binds to BPNSs through non-covalent interactions, and black phosphorus nanosheets (BPNSs) enhance the mechanical stability of the DNA hydrogel through physical interactions with the DNA backbone. The nano-enhanced DNA hydrogel can control the release rate of VEGF, significantly promoting the migration and proliferation of human umbilical vein endothelial cells (HUVECs) and enhancing angiogenesis. Additionally, the synergistic effect of BPNSs and VEGF also accelerates the osteogenic differentiation of BMSCs [169] (Fig. 11D). Furthermore, Liu et al. combined DNA hydrogel with VEGF and nerve growth factor (NGF) to develop a novel delivery system [170]. This system capitalizes on the unique properties of DNA hydrogel to facilitate the dual-phase release of VEGF and NGF. In vitro studies demonstrate that the DNA gel + VEGF/NGF system can sustain the viability of rat Schwann cells (RSCs) (Fig. 11E).

In conclusion, the combination of DNA hybrid hydrogels with cartilage engineering brings great hope for the development of this field. These hydrogels can precisely control cell behavior through customized structures. This regulatory capability can be applied in multiple areas, from protecting encapsulated cells from shear forces to achieving controlled cell release. Besides cell encapsulation, DNA hybrid hydrogels can also serve as multifunctional carriers for therapeutic drugs, delivering medications, cytokines, and exosomes to target sites, thereby enhancing therapeutic efficacy. Furthermore, by using specific aptamers to simulate the ECM, they can promote cell-specific recruitment and differentiation, thus becoming the core of tissue engineering strategies. With their multifunctional capabilities, DNA hybrid hydrogels represent a dynamic toolkit with transformative potential, poised to redefine cartilage engineering and tissue regeneration.

## 6 DNA-encoded dynamic hydrogels for cartilage organoids

The development of cartilage organoids is still at an early stage, yet there have already been successful demonstrations of differentiating various stem cells into cartilage organoids. One significant limitation, however, has been that the biomaterials traditionally used do not adequately simulate the natural environment necessary for chondrocyte survival. DNA hydrogels, characterized by their programmable sequences and adjustable mechanical properties, offer a promising solution by enabling the creation of microenvironments that closely resemble the ECM of cartilage. The application of DNA hydrogels in cartilage tissue construction not only introduces a new material option for cartilage tissue engineering but also suggests new possibilities for the construction of cartilage organoids.

### 6.1 Conventional strategy of cartilage organoids

The construction of cartilage organoids mainly relies on hMSCs, iPSCs, and embryonic stem cells (ESCs). Under appropriate culture conditions, these cells can differentiate into chondrocytes and form 3D organoid structures. In the early stages, scientists encapsulated primary rat rib cells in hollow fibers to induce the formation of cylindrical cartilage organoids [51]. O'Connor and colleagues [171] created cartilage organoid tissue in vitro by continuously exposing iPSCs to chondrogenic growth factors. Some scaffold materials used for organoid construction, such as hyaluronic acid (HA) and PEG, can provide the necessary microenvironment for cell adhesion, growth, and differentiation. For instance, Yang et al. [172] created custom gelatin-based microgels containing HA and hydroxyapatite (HYP). These microgels can self-assemble into cartilage organoid tissue in vivo, thereby promoting cartilage tissue regeneration.

Improvements in constructing cartilage organoids are necessary, especially with the advent of 3D bioprinting technology, which offers significant advancements over traditional methods by allowing precise control over the architecture, composition, and functionality of cartilage implants [173,174]. This technology not only facilitates the creation of patient-specific geometrical shapes but also addresses the unique functional requirements of different cartilage defects, such as providing personalized implants for nasal defects. Furthermore, for effective cell accommodation, the 3D bioprinted materials must closely mimic the natural ECM, providing a conducive physiological environment for in vivo-like cell proliferation and differentiation. Additionally, achieving the desired mechanical properties in cartilage organoids is crucial; bioinks used in 3D printing must have tunable properties to closely resemble the biomechanical environment of natural cartilage, essential for studying cell-matrix interactions [28,175]. Lastly, guiding the differentiation of chondrocytes specifically towards cartilage tissue, rather than bone, necessitates a targeted approach in cartilage regeneration, emphasizing the need for precise biochemical cues and mechanical conditions in the bioprinting process to ensure the successful construction of cartilage organoids.

### 6.2 DNA-encoded bioink for advanced cartilage organoids

**Fig. 12:**
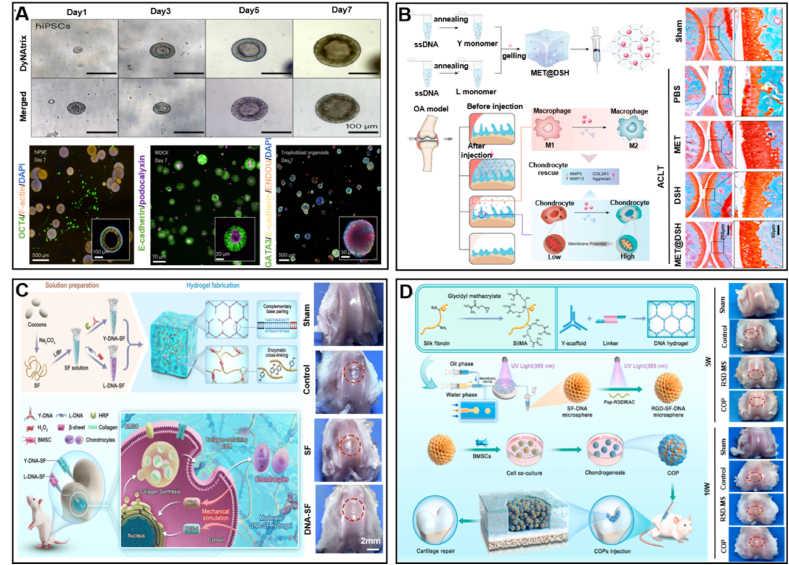
Application of DNA hydrogels in cartilage. (A) DyNAtrix is suitable for use with a range of 3D cell cultures and organoid cultures. Reprinted with permission from Ref. [13]. Copyright 2023, Nature. (B) Preparation and working principle of MET@DSH and in vivo chondrocyte-promoting repair effects. Reprinted with permission from Ref. [177]. Copyright 2023, American Chemical Society. (C) Synthesis of double network DNA-SF hydrogels and schematic illustration of promoting knee cartilage repair. Reprinted with permission from Ref. [39]. Copyright 2024, Royal Society of Chemistry. (D) Synthesis of RGD-SF-DNA microspheres and COP, and schematic illustration of promoting femoral cartilage repair. Reprinted with permission from Ref. [40]. Copyright 2024, Elsevier.

Traditional hydrogels such as collagen, alginate, and PEG are widely used in tissue engineering due to their biocompatibility; however, they have limited mechanical tunability and lack dynamic responsiveness to changes in the cellular microenvironment [176]. These limitations are particularly critical in cartilage organoid applications, where the hydrogel matrix must adapt to mimic the dynamic nature of the native ECM. In contrast, DNA hydrogels offer programmable mechanical properties, including stiffness, elasticity, and degradation rates, which can be precisely controlled to respond to environmental cues. This adaptability makes DNA hydrogels particularly suitable for replicating the native cartilage ECM, supporting chondrogenic differentiation, and promoting more effective tissue regeneration. Additionally, DNA hydrogels can be engineered to incorporate biochemical signals, such as growth factors or peptides, to further enhance cell viability, proliferation, and differentiation, ensuring better integration in vivo. These unique attributes provide DNA hydrogels with significant advantages over conventional hydrogels, making them a promising tool for advanced cartilage organoid development and regenerative cartilage tissue engineering. The previous text introduced DyNAtrix, designed by Peng et al. [13], which can replicate the mechanical properties of living tissues and reproduce the physiological environment in which cells live. As a result, culturing various types of stem cells and human trophoblast organoids using DyNAtrix demonstrated high viability, proliferation, and morphogenesis. It is important to note that trophoblast organoids were resoundingly maintained in DyNAtrix for a period of 3 weeks (Fig. 12A). Attaining this extended period of culture is essential for the advancement of research into numerous other human organoid systems in the future. In 2023, zhang et al. explored a new strategy for treating OA [177]. The authors focused on the potential of metformin (MET) for OA treatment. Although MET has shown promising capabilities, its effectiveness in the joint cavity is limited by rapid clearance and the inability to overcome the severe inflammatory environment, which significantly reduces its therapeutic efficacy. To overcome these challenges, the authors developed a DNA supramolecular hydrogel (DSH) as a sustained drug delivery system for MET. The DSH delivery platform, called MET@DSH, not only extends the retention of MET in the joint cavity from 3 to 14 days, but also actively participates in the reduction of local inflammation through the inherent anti-inflammatory and antioxidant properties of DNA. This comprehensive approach results in the polarization of macrophages from a pro-inflammatory state (M1) to an anti-inflammatory state (M2), diminishing local inflammation, rescuing the decreased mitochondrial membrane potential in chondrocytes, and progressively restoring the joint cartilage to a healthy condition (Fig. 12B). Additionally, Zhou et al. developed a tailored dual-network hydrogel composed of DNA and silk fibroin (SF), where the mechanical properties of the hydrogel were modulated by controlling the concentration of DNA, thereby prompting the formation of β-sheet structures within the SF molecules. Significantly, this dual-network hydrogel creates an advantageous physiological milieu for the chondrogenic differentiation of BMSCs, and it has exhibited capabilities for hastening cartilage regeneration within rat models [39] (Fig. 12C). Recently, building on Zhou et al.'s research on cartilage regeneration with SF-DNA double network hydrogels, Shen et al. created RGD-SF-DNA hydrogel microspheres (RSD-MSs) that can load BMSCs [40]. Initially, SF-DNA double network hydrogel microspheres (SD-MS) were prepared by integrating silk methacrylate (SilMA) with supramolecular DNA hydrogel technology through microfluidic techniques. Afterward, these hydrogel microspheres were surface-modified through photopolymerization with Pep-RGDfKA. Furthermore, BMSCs were implanted into these RSD-MSs to perform in vitro experiments for cartilage induction, resulting in the formation of cartilage organoid precursors (COPs). The findings demonstrate that BMSCs display enhanced cellular viability in COPs, and that COPs notably facilitate the regeneration of cartilage (Fig. 12D). Therefore, this study also provides new insights into the application of DNA hydrogels for cartilage organoids. From this, it is evident that using DNA hydrogels as bioinks for 3D printing to construct cartilage organoids indeed holds promise as a superior and effective strategy [176].

## 7 Conclusion and future perspective

OA inflicts a substantial burden on patients, with one contributing factor being the degeneration of joint cartilage due to cartilage defects. Unfortunately, cartilage has a markedly limited ability to regenerate, and existing therapeutic approaches for addressing cartilage defects are fraught with various limitations. In recent years, the continuous development of organoid technology has increasingly attracted attention. Therefore, to address this issue, 3D printing biotechnology can be employed to construct cartilage organoids in vitro for the repair of cartilage defects. The critical aspect of 3D bioprinting involves choosing the correct bioink, specifically a dynamic bioink that can accurately emulate the properties of the ECM. However, traditional bioinks used for 3D printing are mostly static, making it challenging to meet the requirements of the cartilage cell living environment.

DNA, as a biological macromolecule, possesses unique sequence programmability, enabling it to form predictable complex structures, perform computational operations, or control biological functions [178]. Crosslinking DNA with dynamic hydrogels allows the DNA hydrogel to simultaneously possess the functionalities of both. Compared with hydrogels derived from natural polymers, DNA hydrogels exhibit distinct programmability attributed to Watson-Crick base pairing [179,180]. Moreover, the intrinsic flexibility of DNA molecules grants DNA hydrogels the ability for precise structural customization and adjustable mechanical performance. DNA hydrogels also exhibit inherent biocompatibility and biodegradability, showcasing significant potential in cell culture applications [181]. Therefore, utilizing DNA hydrogels to mimic the cartilage ECM is an ideal choice. This review elucidates the strategy of using DNA hydrogels as bioinks for 3D printing cartilage-like organoids to treat cartilage defects.

DNA hydrogels have the potential to merge with biotechnological advancements and theranostic applications. For example, leveraging the ongoing progress in DNA nanotechnology [182], they can be utilized to create highly sensitive and specific biological sensors designed to track biochemical transformations in cartilage tissue. These sensors can provide accurate biomarker detection in the early stages of cartilage degeneration or injury, aiding in early diagnosis and treatment. Furthermore, the high plasticity and responsiveness to environmental stimuli of DNA hydrogels make them suitable for use as components of actuation devices, such as artificial muscles or other devices capable of mimicking the behavior of biological tissues. Additionally, DNA hydrogels have also made advancements in the field of gene editing and therapy. With the development of gene editing technologies such as CRISPR, DNA hydrogels could play a role in precisely delivering editing complexes to target cells. They can also serve as carriers for the safe and effective transfer of therapeutic genes into patients [183,184].

**Fig. 13:**
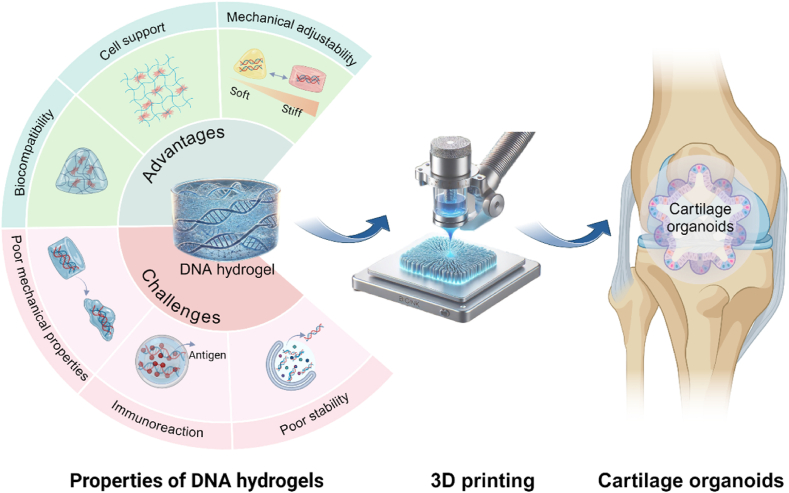
Prospects and challenges of DNA hydrogels. Created with BioRender.com.

Although DNA hydrogels have numerous advantages, there are still some challenges that need to be addressed before their widespread application. For instance, the mechanical strength and stability of DNA hydrogels are relatively low, which limits their applications in scenarios requiring pressure resistance, such as load-bearing scaffolds in tissue engineering. However, this issue can be addressed by crosslinking DNA hydrogels with other polymers to form multi-network DNA hydrogels [135]. Additionally, he degradation of DNA hydrogels is another issue that needs to be considered. Typically, the degradation products of DNA hydrogels are considered safe, but for long-term applications or when used in large doses, it is necessary to ensure that the degradation products do not cause adverse biological reactions. For instance, inappropriate degradation of DNA hydrogels within the body may result in the formation of small, short-stranded DNA molecules. These molecules have the potential to activate the STING pathway, leading to the enhanced generation and release of type I interferons and additional inflammatory cytokines, thus provoking an immune response [185]. Recently, Zhou and colleagues have mitigated the inherent biodegradability of natural DNA by substituting D-DNA with its mirror image enantiomer. The resulting nuclease-resistant L-DNA hydrogels possess entirely identical nucleotide sequences and inherently the same network topology and mechanical properties. Furthermore, the stiffness of these hydrogel DNA hydrogels can be adjusted, allowing them to serve as analogs for 3D ECM [186]. Furthermore, current research on DNA hydrogels is primarily focused on a limited type of cells, such as MSCs. Hence, to more effectively simulate and reconstruct the cellular habitat, it is necessary to conduct further studies on the use of DNA hydrogels for the culture of diverse cell types, with the aim of fabricating a broader array of tissues and organoids structures. Additionally, when DNA hydrogels are implanted in the body, they may be recognized as foreign substances by the immune system, potentially triggering an immune response. This could lead to inflammation, tissue rejection, or other immune-related reactions. Finally, the cost associated with the DNA molecules needed for fabricating DNA hydrogels is typically substantial, potentially hindering their economic effectiveness for mass production and widespread use. Addressing this problem may involve refining synthesis techniques and employing more affordable materials, among other approaches (Fig. 13).

In the near future, scientists should further explore the application of DNA hydrogels as 3D culture scaffolds for constructing cartilage organoids. These cartilage organoids can serve as models for studying diseases and defects in vitro, providing valuable insights into disease mechanisms and drug actions, as well as enabling drug screening. Furthermore, using cartilage organoids to evaluate the regenerative and reparative functions of biomaterials, instead of animal models, can better address ethical concerns associated with animal testing.

In summary, DNA hydrogels, with their unique sequence programmability and tunable mechanical properties, have become an ideal biomaterial for constructing cartilage organoids. They offer a novel strategy for addressing cartilage damage and degeneration, with their potential applications in the field of biomedicine continuing to grow. Hence, we expect that in the near future, through ongoing scientific exploration and technological innovation, there will be promising advancements in the development of more precise and functional solutions for cartilage tissue engineering, thereby providing patients with more effective treatment options.

## CRediT authorship contribution statement

**Ziyu Chen:** Writing – original draft, Methodology, Formal analysis, Data curation, Conceptualization. **Hao Zhang:** Writing – review & editing, Visualization, Validation, Resources, Methodology, Conceptualization. **Jingtao Huang:** Visualization, Validation, Supervision, Conceptualization. **Weizong Weng:** Visualization, Supervision, Conceptualization. **Zhen Geng:** Visualization, Supervision, Investigation. **Mengmeng Li:** Supervision, Methodology, Conceptualization. **Jiacan Su:** Supervision, Resources, Methodology, Investigation.

## Declaration of competing interest

The authors declare that they have no known competing financial interests or personal relationships that could have appeared to influence the work reported in this paper.

## Data Availability

No data was used for the research described in the article.
